# The Effect of Grain Position on Genetic Improvement of Grain Number and Thousand Grain Weight in Winter Wheat in North China

**DOI:** 10.3389/fpls.2018.00129

**Published:** 2018-02-07

**Authors:** Fan Feng, Yunliang Han, Shengnan Wang, Shaojing Yin, Zhenyu Peng, Min Zhou, Wenqi Gao, Xiaoxia Wen, Xiaoliang Qin, Kadambot H. M. Siddique

**Affiliations:** ^1^College of Agronomy, Northwest A&F University, Yangling, China; ^2^School of Biological and Chemical Engineering, Panzhihua University, Panzhihua, China; ^3^The UWA Institute of Agriculture and School of Agriculture and Environment, The University of Western Australia, Perth, WA, Australia

**Keywords:** winter wheat, genetic improvement, grain position, grain weight, grain number

## Abstract

Genetic improvements have significantly contributed to wheat production. Five wheat cultivars—widely grown in north China in the 1950s, 1990s, or 2010s—were grown in field experiments conducted in the 2014–2015 and 2015–2016 growing seasons. This study evaluated the genetic progress in wheat grain yield and its related traits in north China and explored how breeding and selection have influenced grain numbers and weights within spikelets in the past 60 years. The results showed that the significant increases in grain yield in the past 60 years were mainly due to increases in grain number per spike and grain weight, while spike number per m^2^ has not changed significantly. Improvements in thousand grain weight (TGW) from the 1950s to 2010s have occurred at four grain positions (G1 to G4). The relative contribution of G4 to TGW increased over time, but was much less than the contributions of G1, G2, and G3. Indeed, the average grain weight at G4 was much less than that of 1000 grains. The increase in grain number per spike since the 1950s was mainly due to an increase in grain number at G1, G2 and G3, with the relative contribution of grain position to grain number being G1 > G2 > G3 > G4. Dwarfing genes increased grain number per spike and grain number at G3 and G4, but not TGW. In future, yields could be boosted by enhancing grain weight at G4 and grain number at G3 and G4, while maintaining those at G1 and G2.

## Introduction

Wheat is an important crop throughout the world (Morgounov et al., 2010; Manès et al., 2012; Beche et al., 2014). There have been significant genetic improvements in wheat yields in the past decades (Siddique et al., 1989a,b; Zhou et al., 2007a; Xiao et al., 2012; Sun et al., 2014). In China, the average wheat yield has increased from less than 1 t ha^-1^ in 1949 to 5 t ha^-1^ in 2013, with total production of 126 Mt in 2014 (Qin et al., 2015). With the increasing population pressure and subsequent demand for agricultural products, China will need 776 Mt grain by 2030 to feed its people, a 36% increase from 2014 (Li et al., 2014).

Breeding and selection has significantly affected yield and its components, including the introduction of the Rht dwarfing gene to wheat breeding programs in the 1960s, reduced plant height, higher grain numbers per spike, and higher yields in many wheat-growing regions (Waddington et al., 1986; Perry and D’Antuono, 1989; Slafer et al., 1994; Royo et al., 2007;

Álvaro et al., 2008; Battenfield et al., 2013). Most studies have attributed the increased wheat yields in past decades to increases in grain number per spike (Siddique et al., 1989a), thousand grain weight (TGW), or both (Donmez et al., 2001; Zhou et al., 2007a; Zheng et al., 2011; Xiao et al., 2012; Zhang et al., 2016). However, few studies have paid attention to changes in TGW and grain number per spike at different grain positions.

A wheat spike comprises spikelets, each with several florets (Siddique et al., 1989b; Cui and Guo, 2010). Within a spikelet, individual grain weights and fruiting efficiencies vary due to uneven development (Chen et al., 2007; Ferrante et al., 2015; Guo and Schnurbusch, 2015). Grain numbers and weights differ between and within spikelets (Miralles and Slafer, 1995; He et al., 2000; Qu et al., 2009; Li Y. et al., 2016). The middle spikelets tend to have more and heavier grains than the basal and top spikelets, as is the case for grain positions G1 and G2 compared to G3 and G4 (Loss et al., 1989; He et al., 2000; Qu et al., 2009). Spikelet number, grain weight and grain number per spikelet have a significant effect on TGW and grain number per spike (Green et al., 2012; Wu et al., 2014). There are few published studies on the effect of grain position on TGW or grain number per spike over time in winter wheat in China; more work is needed to enrich our knowledge of how breeding and selection (including the dwarfing gene) influence grain numbers and weights within spikelets.

The most important winter wheat region in China is in the north (Zhang et al., 2007). We conducted field experiments over two successive years using five wheat varieties released in different decades to evaluate the genetic progress of wheat grain yield and related traits through modern breeding in north China and to explore how breeding and selection have influenced grain numbers and weights within spikelets in the past 60 years.

## Materials and Methods

### Experimental Design

**FIGURE 1:**
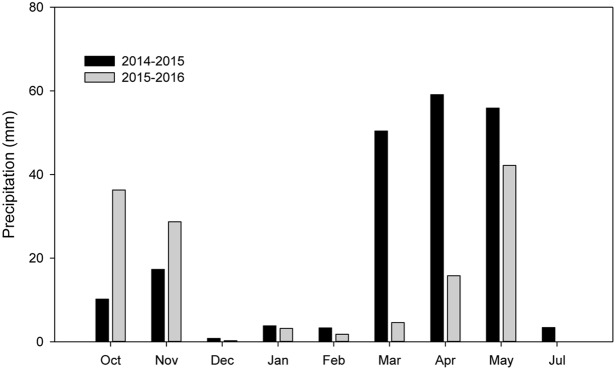
Monthly precipitation distribution during the winter wheat growing seasons at Doukou Experimental Station, Shaanxi Province, China in 2014–2015 and 2015–2016.

The experiments were conducted during the 2014–2015 and 2015–2016 growing seasons in the same field at Doukou Experimental Station (Jingyang, Shaanxi Province, China; 34°36′ N, 108°52′ E; altitude 427.4 m). The experimental site is in a typical, dry, semi-humid area in northwest China (Li C. et al., 2016). The 0–20 cm soil layer contains 15.71 g kg^-1^ organic matter, 1.21 g kg^-1^ total nitrogen, 22.57 mg kg^-1^ available phosphate, and 249.48 mg kg^-1^ available potassium. In both seasons, fertilizer was applied before sowing at a rate of 225 kg ha^-1^ N, 120 kg ha^-1^ P_2_O_5_, and 225 kg ha^-1^ KCl. Precipitation during the wheat growing season was above average in 2014–2015 (204.20 mm) and below average in 2015–2016 (132.87 mm) (**Figure 1**).

Five bread wheat cultivars, released in the 1950s, 1990s or 2010s, were selected for this study (**Table 1**). Bima1 represented the first cultivar replacement in the 1950s (the reason for wide adoption of Bima1 was stronger resistance to stripe rust), Xinong881 and Shan229 represented the last cultivar replacement in the 1990s (more resistant to powdery mildew and stripe rust, lodging resistance and higher yields), and Shanmai139 and Zhoumai26 are new cultivars released in 2011 and 2012 (lodging resistance and higher yields). The trial was arranged in a completely randomized design with three replications. Each 5 m^2^ plot consisted of 10 rows (2 m long × 0.25 m wide). The experiments were sown on 15 October in 2014 and 2015 at a rate of 230 kernels m^-2^ and harvested on 4 June 2015 and 7 June 2016, respectively. Maize (*Zea mays* L.) was the previous crop. According to local cultivation practices, furrow irrigation (1500 t ha^-1^) was applied at the tillering stage (before winter, GS25) (Zadoks et al., 1974). Insects and diseases were controlled by spraying the recommended fungicides and insecticides. Weeds were removed by hand throughout the growing seasons. Bamboo sticks and plastic ropes tied to the sticks were used to prevent tall plants from lodging so that the maximum yield potential could be reached.

**Table 1:** Year of release, origin, decade of promotion, pedigree, and dwarf gene of the five wheat cultivars in this study.

Cultivar	Year of release	Origin	Decade of promotion	Pedigree	Dwarf gene(s)^a^
Bima1	1948	Shaanxi, China	1950s	Mazhamai/Florence	–
Xinong881	1992	Shaanxi, China	1990s	Xiaoyan6/Xinong65/83(2)-3-3	*Rht-D1b*
Shan229	1993	Shaanxi, China	1990s	Shaan7853/80356	*Rht-B1b+Rht8*
Shanmai139	2011	Shaanxi, China	2010s	Xiaoyan22/94156/N9134	*Rht-B1b*
Zhoumai26	2012	Henan, China	2010s	Zhoumai24/Zhoumai22	*Rht-D1b+Rht8*

*^a^The presence of dwarfing genes in five bread wheats was obtained from State Key Laboratory of Crop Stress Biology for Arid Areas in College of Agronomy, Northwest A&F University, China.*

### Plant Sampling and Measurements

Two central rows (each 1 m long) from each plot were harvested at maturity (GS92). Spike number per m^2^, grain number per spike, TGW (after being dried for 24 h at 60°C) and yield were calculated from the samples. Anthesis date was recorded according to Zadoks et al. (1974). Plant height was measured at maturity (GS92).

**FIGURE 2:**
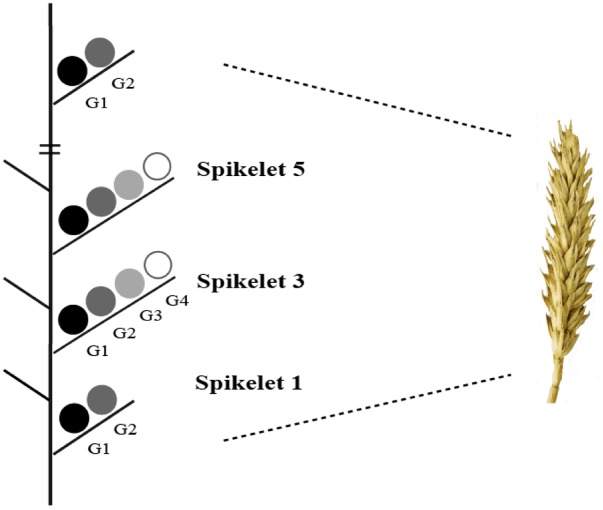
Schematic diagram indicating grain positions in different spikelets (spikelet 1 is the lowest). Adapted from Areche and Slafer (2006).

To determine the dry weight of individual grains at different positions within a spike, 30 spikes were harvested at maturity (GS92) from each plot. Each spikelet and grain was weighed separately after being dried for 24 h at 60°C. Grain positions on each spikelet are indicated in the schematic diagram (**Figure 2**). The number of grains per spikelet and at G1 to G4 was counted.

### Statistical Analysis

Analysis of variance was carried out for each growing season using the General Linear Model procedure in SAS (SAS Institute, 2003). Multiple comparisons between the treatments used the Least Significant Difference test (LSD_0.05_). All figures were created with Sigmaplot 11.0.

The relative contribution of grain at each grain position was calculated using the following equation:

$${\text{y}}_{\text{i}}=\frac{{\text{x}}_{\text{i}}}{{\text{∑}}_{\text{i}=1}^{4}{\text{x}}_{\text{i}}}\times 100\%$$

where y_i_ is the relative contribution of grain number or grain weight at a certain grain position, and x_i_ is grain number or grain weight at the same grain position (i ranged from 1 to 4, representing G1, G2, G3, G4, respectively).

## Results

### Yield and Yield Components

Grain yield increased significantly with year of release (**Table 2**) from 4.02 t ha^-1^ for Bima1 released in the 1950s to 9.35 t ha^-1^ for Shanmai139 released in 2011 during the 2014–2015 growing season, and from 3.56 t ha^-1^ for Bima1 to 6.83 t ha^-1^ for Zhoumai26 released in 2012 during the 2015–2016 growing season. Significant genetic gains were also observed for TGW and grain number per spike. TGW ranged from 26.21 g for Bima1 to 38.88 g for Zhoumai26 in 2014–2015 and from 26.72 g to 38.68 g for the same cultivars in 2015–2016. Grain number per spike ranged from 35.00 for Bima1 to 47.15 for Shanmai139 in 2014–2015, and from 32.13 to 43.63 for the same cultivars in 2015–2016. In contrast, spike number per m^2^ did not change over time. Compared with Bima1 released in the 1950s, the other four cultivars (released in 1990s and 2010s) had significantly lower plant heights due to the presence of dwarfing genes *Rht-B1b*, *Rht-D1b*, and *Rht-8*. Anthesis date did not significantly differ between the five wheat cultivars.

**Table 2:** Analysis of variance for yield and related traits of each wheat cultivar during the 2014–2015 and 2015–2016 growing seasons.

Cultivar	Yield (t ha^-1^)		Spike number per m^2^		TGW (g)		Grain number per spike		Plant height (cm)		Anthesis date (days)	
	2014–2015	2015–2016	2014–2015	2015–2016	2014–2015	2015–2016	2014–2015	2015–2016	2014–2015	2015–2016	2014–2015	2015–2016
Bima1	4.02c	3.56c	633.00ab	493.00a	26.21c	26.72d	35.00c	32.13c	151.3a	121.7a	197.0a	191.7b
Xinong881	6.25b	5.40b	584.67ab	476.67a	35.21b	35.51bc	38.02bc	36.13bc	86.7c	68.3bc	194.7b	191.3b
Shan229	6.87b	5.17b	668.67a	490.00a	34.84b	34.26c	40.52b	38.35b	95.3b	75.3b	197.0a	191.7b
Shanmai139	9.35a	6.30ab	607.33ab	457.33a	38.56a	37.38ab	47.15a	43.63a	81.7c	69.3bc	196.3ab	193.0a
Zhoumai26	8.52a	6.83a	560.00b	478.67a	38.88a	38.68a	46.83a	43.18a	82.3c	66.3c	197.0a	191.7b
*F*(test)	^∗∗^	^∗∗^	ns	ns	^∗∗^	^∗∗^	^∗∗^	^∗∗^	^∗∗^	^∗∗^	ns	ns

*Means followed by the same letter within a column are not significantly different according to LSD_0.05_. ^∗∗^P = 0.01; ns, not significant.*

**FIGURE 3:**
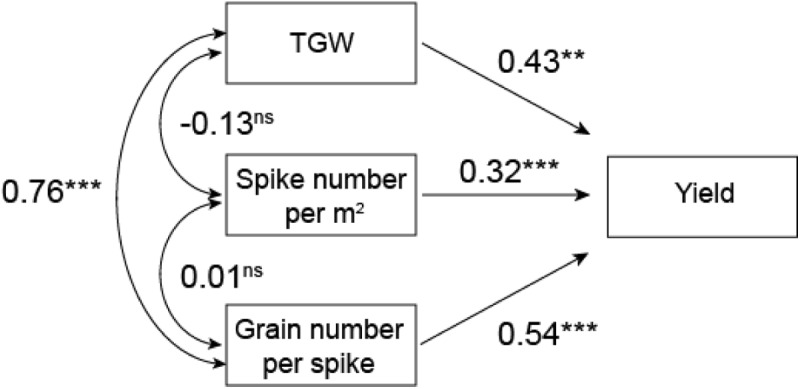
Path analysis of TGW, spike number per m^2^ and grain number on yield in five wheat cultivars. ^∗∗∗^*P* = 0.001; ^∗∗^*P* = 0.01; ns, not significant.

Path analysis showed that grain number per spike had the greatest significant direct effect on yield with indirect positive effects of TGW (0.33). TGW had the second highest direct effect on yield with indirect effects of grain number per spike (0.41) (**Figure 3**).

### Individual Grain Weights at Different Grain Positions within a Spikelet

**FIGURE 4:**
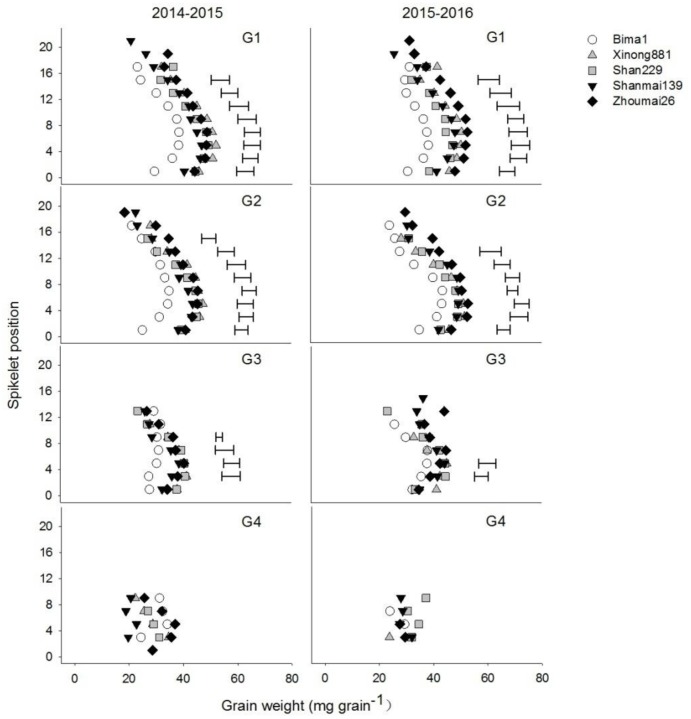
Individual grain weights at different grain positions (G1–G4) on each spikelet in five wheat cultivars released in different decades and grown in 2014–2015 and 2015–2016. Each point represents the average value of two spikelets. Horizontal bars represent the LSD at *P* = 0.05.

Grain weights at G1, G2, and G3 increased from the bottom to middle spikelets but decreased from the middle to top spikelets (**Figure 4**). Grain weight at G1 and G2 increased significantly with year of release from the 1st spikelet to the 14th or 16th spikelet. Grain weight at G3 differed significantly between cultivars from the 3rd to the 6th spikelet, and there was an increasing trend from the 1950s to 2010s. Grain weight at G4 did not differ between cultivars (**Figure 4**).

### Grain Weight and Grain Number in Different Spikelets and Grain Positions

**FIGURE 5:**
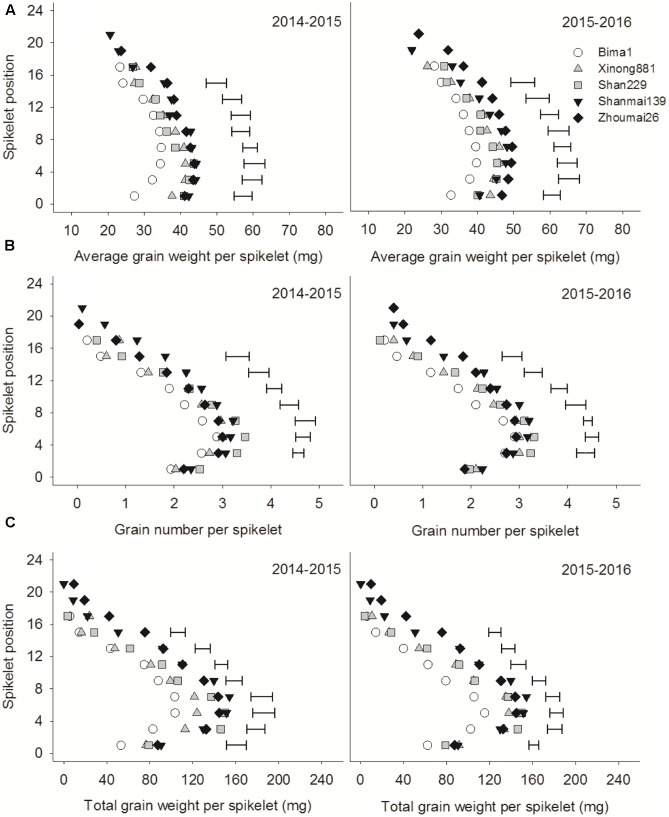
Average grain weight **(A)**, grain number **(B)**, and total grain weight **(C)** in different spikelets of five wheat cultivars released in different decades and grown in 2014–2015 and 2015–2016. Each point represents the average value of two spikelets. Horizontal bars represent the LSD at *P* = 0.05.

Average grain weight, grain number, and total grain weight per spikelet increased from the bottom to middle spikelets and then decreased from the middle to top spikelets (**Figure 5**). Shanmai139 and Zhoumai26 released in the 2010s had more spikelets than the older cultivars. Grain number per spikelet, from the 3rd to 16th spikelet, increased significantly with year of release. Total grain weight and average grain weight per spikelet followed a similar trend, gradually increasing from the 1st to 16th spikelet (**Figure 5**).

**FIGURE 6:**
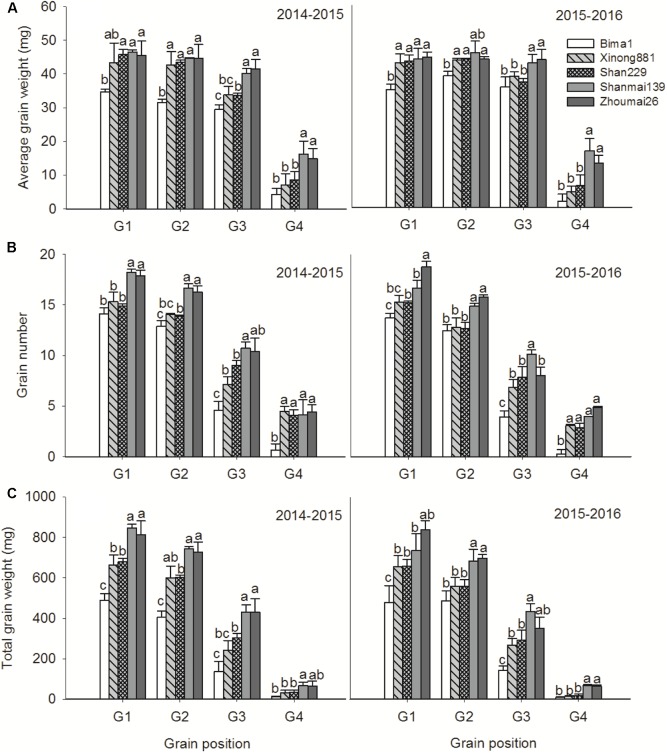
Average grain weight **(A)**, grain number **(B)**, and total grain weight **(C)** at each grain position in five wheat cultivars released in different decades and grown in 2014–2015 and 2015–2016. The data represent means ± SE. Different lowercase letters above the bars denote significant differences between cultivars.

Average grain weight increased significantly at G1 and G2 from the 1950s to 1990s and at G3 and G4 from the 1990s to 2010s. Grain numbers at G1, G2, and G3 increased from the 1950s to 2010s; G4 increased significantly from the 1950s to 1990s but did not change from the 1990s to 2010s (**Figure 6**). Total grain weights at G1, G2, and G3 gradually increased from the 1950s to 2010s, and G4 increased significantly from the 1990s to 2010s but did not change from the 1950s to 1990s in either growing season (**Figure 6**).

### Relative Contribution of Each Grain Position to Grain Weight and Grain Number Per Spike

**FIGURE 7:**
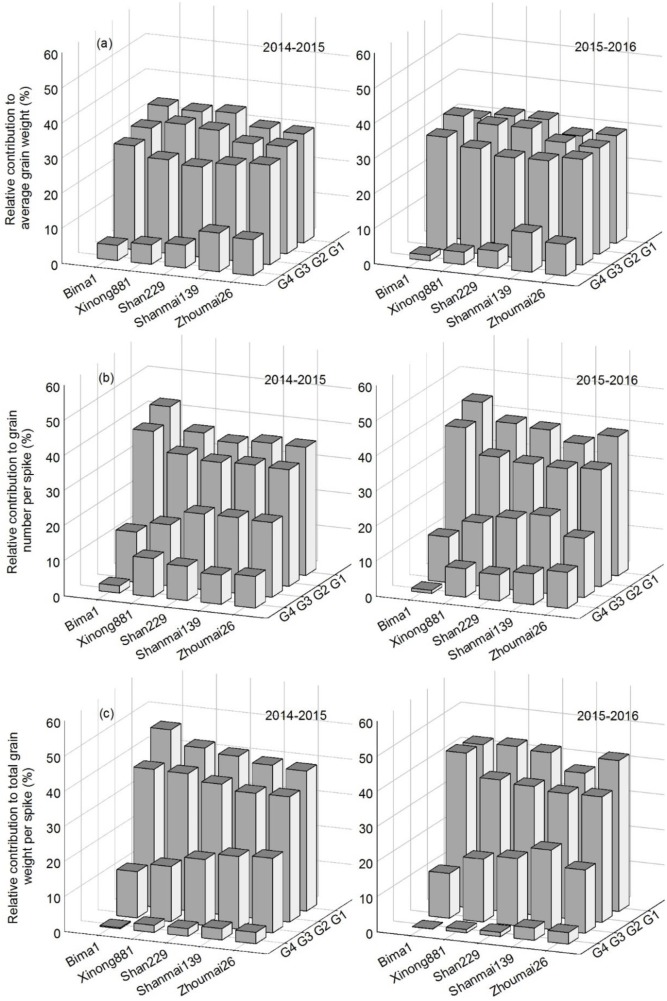
The relative contribution of grain at each grain position to grain weight **(a)**, grain number per spike **(b)** and total grain weight per spike **(c)** in five wheat cultivars released in different decades and grown in 2014–2015 and 2015–2016.

For average grain weight, G4 had the lowest relative contribution of the four grain positions, which increased with year of release; the relative contribution increased from G4 to G3 to G2 and G1. For grain number per spike and total grain weight per spike, the relative contribution increased from G4 to G3 to G2 to G1 and with year of release from the 1950s to 2010s (**Figure 7**).

## Discussion

The yield of winter wheat has increased in China in recent decades (Zhang et al., 2016). This study confirmed that wheat yields in the northern plain of China have significantly increased with year of cultivar release (**Table 2**). Studies have shown that these wheat yield improvements are primarily due to increases in grain number per spike (Donmez et al., 2001; Zheng et al., 2011) and TGW (Morgounov et al., 2010; Zhang et al., 2016), which was confirmed in the present study (**Figure 3**). Grain number per spike had the most significant direct effect on yield, and mean TGW had the second highest direct effect on yield, which agrees with Bustos et al. (2013) and García et al. (2013). We found no apparent change in spike number per m^2^, which agrees with the findings of Zheng et al. (2011) and Zhang et al. (2016) in the Henan Province, but disagrees with those of Zhou et al. (2007b) and Xiao et al. (2012), who reported a reduction in spike number per m^2^ over time. Studies have shown that the presence of dwarfing genes, including *Rht-B1b*, *Rht-D1b* and *Rht-8*, are associated with higher grain numbers per spike, relatively lower average grain weights, and no change in spike number per m^2^, which increased grain yield (Youssefian et al., 1992; Miralles and Slafer, 1996; Royo et al., 2007; Uzik and Zofajova, 2007; Álvaro et al., 2008; Rebetzke et al., 2011). In the current study, four cultivars with dwarfing genes had higher grain numbers and grain yield than Bima1, which agrees with previous studies. However, these four cultivars had higher average grain weights than Bima1, which differs from previous studies, suggesting that dwarfing genes are not the major genes affecting grain weight between cultivars; the different results are likely due to the fact that most previous studies used near-isogenic lines (Youssefian et al., 1992; Miralles and Slafer, 1996; Flintham et al., 1997; Miralles et al., 1998; Rebetzke et al., 2011), while our study used cultivars with different pedigrees. Furthermore, there was no clear trend on the effect of different dwarfing genes on grain number, grain weight, and grain yield between the four cultivars with dwarfing genes in this study.

Breeders have long-focused on TGW as a vital yield component for winter wheat, increasing over time as yields improved (Donmez et al., 2001; Morgounov et al., 2010; Zheng et al., 2011). However, most studies have not considered the effects of grain at different grain positions on TGW. Our study showed that G1 and G2 had similar grain weights, which were higher than that at G3 and G4 in every tested cultivar, and agrees with previous reports (Pan et al., 2005; Qu et al., 2009; Li Y. et al., 2016). TGW improvements from the 1950s to 2010s varied between grain position, with average grain weights at G1, G2, G3, and G4 in cultivars released in the 2010s significantly higher than in Bima1 released in the 1950s. The relative contribution of grain weight to TGW at G1, G2, and G3 has remained fairly stable over time. While the relative contribution of grain weight to TGW at G4 has increased over time, it is much less than at G1, G2, and G3. Average grain weight at G4 is also much less than TGW. Thus, increasing grain weight at G4, while maintaining those at G1, G2 and G3, could increase TGW.

Studies have shown a significant positive correlation between grain number per spike and spikelet number per spike (Donmez et al., 2001; Green et al., 2012; Wu et al., 2014). In this study, Shanmai139 and Zhoumai26 (released in the 2010s) had more spikelets than the other cultivars in both growing seasons (**Figures 4**, **5**). In addition, grain numbers at G1, G2, and G3 increased from the 1950s to 2010s but did not change at G4 from the 1990s to 2010s (**Figure 6**). The relative contribution of grain position to grain number was G1 > G2 > G3 > G4. Therefore, we conclude that the increased grain number per spike is due to increases in average grain numbers at G1, G2, and G3. More grains per spike and higher yields in wheat could be achieved by improving grain numbers at G3 and G4 without decreasing grain weights at G1 and G2. In this study, the four cultivars with dwarfing genes had significantly higher grain numbers at G3 and G4 than Bima1, which is consistent with previous findings where dwarfing genes affected grain numbers at G3 and G4 by increasing fertile florets, resulting in more grains per spike (Miralles and Slafer, 1996; Flintham et al., 1997; Miralles et al., 1998; Rebetzke and Richards, 2000).

Studies have shown that grain weight per spike has greatly contributed to genetic improvements in wheat yield (Zhou et al., 2007a,b; Zheng et al., 2011; Xiao et al., 2012), which was confirmed in this study. Total grain weights at G1, G2, G3, and G4 increased from the 1950s to 2010s with no apparent change in spike number per m^2^. While grain numbers and grain weights at the four grain positions increased at different rates from the 1950s to 2010s, the total grain weight gradually increased, which greatly improved grain yield. Grain number and grain weight per spikelet differ between spikelet and grain positions (He et al., 2000; Qu et al., 2009; Li Y. et al., 2016). Our results showed that grain number, average grain weight, and total grain weight from the bottom to the top spikelets showed parabolic changes, indicating that the middle spikelets had a significant advantage for priority development than the bottom and top spikelets, which agrees with previous studies (He et al., 2000; Guo and Schnurbusch, 2015; Li Y. et al., 2016). Breeding has increased grain number and grain weight in the bottom and middle spikelets of modern cultivars, but only grain number in the upper spikelets (**Figures 4**, **5**). More attention should be paid to the upper spikelets of wheat in future.

## Conclusion

Dwarfing genes have increased grain numbers per spike and grain numbers at G3 and G4, but not reduced TGW as observed in previous studies. Improvements in TGW from the 1950s to 2010s are related to grain weights at four grain positions, with the relative contribution of G4 increasing over time, but contributing much less to grain weight than G1, G2, and G3. The increase in grain number per spike since the 1950s was mainly due to increased grain numbers at G1, G2 and G3, with the relative contribution of grain position to grain number being G1 > G2 > G3 > G4. Genetic improvements in wheat yield have mainly resulted from increases in TGW and grain number per spike over time. In future, grain yields could be boosted by increasing grain weights at G4 and grain numbers at G3 and G4, while maintaining both at G1 and G2.

## Author Contributions

FF and XQ designed the study, performed the experiment. YH, XQ, XW, and KS collected and analyzed the data. All authors participated in the experimental design and manuscript writing.

## Conflict of Interest Statement

The authors declare that the research was conducted in the absence of any commercial or financial relationships that could be construed as a potential conflict of interest.
